# Editorial: Luteal Phase Support for Assisted Reproduction

**DOI:** 10.3389/frph.2021.759122

**Published:** 2021-12-13

**Authors:** Shevach Friedler, Ido Ben-Ami

**Affiliations:** ^1^Barzilai University Medical Center, Ashkelon, Israel; ^2^Shaare Zedek Medical Center, Jerusalem, Israel

**Keywords:** luteal phase, IVF (*in vitro* fertilization), luteal phase deficiency, luteal phase support, progesterone

There is an unchallengeable consensus regarding the need for luteal phase support in IVF, due to the interference of the ovarian activity of the corpus luteum after COH or the lack of it during HRT for FET. It is remarkable that even though luteal support is being prescribed for decades, remarkably there is no consensus yet concerning which medication specifically to be used, at which dose, and for how long. To express the notion that the same outcome can be reached by many methods the author wanted to use the old proverb of “There's **more than one way** to skin a cat,” however, as today this proverb sounds to the author as politically problematic it may be preferable to use another old proverb “all roads lead to Rome.”

As the search for the ideal method is still ongoing and the field is still open for debate, we present on the distinguished platform of Frontiers in Reproduction, a collection of four interesting articles, touching our subject matter of luteal support in IVF from different angles.

A comprehensively written review by Tesarik et al., depicts the scientific basis for the understanding of the pathophysiology of luteal phase insufficiency including suboptimal corpus luteum secretion and/or uterine dysfunction, leading to the need of the various ways of luteal support aiming to cure these two major aspects. They highlight the differences between the cases of progesterone deficit and those with normal progesterone levels. They clarify the justification behind the various methods used for luteal support which should be given until the luteoplacental shift occurs taking over the function of the corpus luteum.

Progesterone, the main agent that is necessary to provide luteal support may be administered by many routes, and we present the latest new ways for its administration, subcutaneous and oral. More options mean more ways to find the best solution for our patients considering their individual preferences. Conforti et al. present a well-performed first systematic review regarding the subcutaneous use of water-soluble progesterone injection for luteal support after COH for IVF, fresh and frozen, or IUI. It shows the advantages, efficiency, and limitations of the current knowledge regarding its use in comparison with intravaginal administration. Drakopoulos et al. present an overview of the current knowledge regarding the orally administered dyhydrogesterone, an old medication that may offer a new and preferable way of luteal support.

Finally, the last manuscript by Mizrachi et al. summarizes the current evidence-based knowledge about the optimal length of luteal support that should be recommended. It shows the lack of sufficiently good quality evidence on this topic in the current literature, challenging future research on this topic. Perhaps, we should pay attention to the findings presented by Di Guardo et al. (1), that even evidence-based facts do not necessarily influence the daily practice of many colleagues in the field of reproductive medicine, when it comes to the specific luteal phase protocol they advise their patients, indicating to my mind that more good quality reach on this topic is ardently awaited.

## Author Contributions

The author confirms being the sole contributor of this work and has approved it for publication.

## Conflict of Interest

The authors declare that the research was conducted in the absence of any commercial or financial relationships that could be construed as a potential conflict of interest.

## Publisher's Note

All claims expressed in this article are solely those of the authors and do not necessarily represent those of their affiliated organizations, or those of the publisher, the editors and the reviewers. Any product that may be evaluated in this article, or claim that may be made by its manufacturer, is not guaranteed or endorsed by the publisher.
